# Talk of the Town mobile app platform: New method for engaging family in STEM learning and research in homes and communities

**DOI:** 10.3389/fpsyg.2023.1110940

**Published:** 2023-01-26

**Authors:** Jonathan F. Kominsky, Igor Bascandziev, Patrick Shafto, Elizabeth Bonawitz

**Affiliations:** ^1^Department of Cognitive Science, Central European University, Vienna, Austria; ^2^Graduate School of Education, Harvard University, Cambridge, MA, United States; ^3^Department of Mathematics and Computer Science, Rutgers University, Newark, Newark, NJ, United States

**Keywords:** informal learning, parent–child interaction, methods, research tools, developmental psychology, education

## Abstract

Children do not just learn in the classroom. They engage in “informal learning” every day just by spending time with their family and peers. However, while researchers know this occurs, less is known about the science of this learning—how this learning works. This is so because investigators lack access to those moments of informal learning. In this mini-review we present a technical solution: a mobile-based research platform called “Talk of the Town” that will provide a window into children’s informal learning. The tool will be open to all researchers and educators and is flexibly adaptable to these needs. It allows access to data that have never been studied before, providing a means for developing and testing vast educational interventions, and providing access to much more diverse samples than are typically studied in laboratories, homes, and science museums. The review details the promise and challenges associated with these new methods of data collection and family engagement in STEM learning sciences.

## 1. Introduction

“Informal learning” is loosely defined as learning that occurs outside the classroom or structured educational tasks. Researchers who study the science of learning and STEM education are well aware that informal learning occurs and that it is a critical contributor to children’s knowledge and competence (Falk et al., 2007; Powell and Peet, 2008; Jones et al., 2013; Morris et al., 2019). However, informal learning is intrinsically difficult to study. By definition it cannot be studied in the classroom. It occurs, often unpredictably, through media content, social interactions with peers, self-guided exploratory play, and—as in the focus of this paper—it can occur in natural interactions between children and parents or caregivers as they go about their everyday lives.

There have been several different kinds of efforts to capture informal learning between parents and children in various contexts. One approach has been to study informal learning in the context of science museums (Callanan, 2012). These studies often involve recording of children’s interactions with their caregivers at specific exhibits, with the goal of studying what children learn from those exhibits and how they engage with them (e.g., Thomas and Anderson, 2013). However, these studies face a number of important limitations. The first and most obvious is that they are restricted to the exhibit in question. In addition, the pool of participants is restricted to families in a given area who visit that museum, which provides a somewhat restricted population that may not be fully representative of the breadth of informal learning experiences (though more representative than most in-lab studies; Callanan, 2012).

A second approach is to study informal learning through parent–child interactions in the home with intensive at-home recording studies, using tools like LENA (Ganek and Eriks-Brophy, 2018) or corpus studies based on previous at-home recordings (e.g., using the CHILDES database, MacWhinney, 2000; or Databrary, Dressler, 2015). This approach circumvents the topic-specificity of museum-based studies, but is still limited by a likely non-representative population and challenges in isolating relevant data. With hundreds or thousands of hours of recordings, identifying relevant segments for informal learning about a specific topic is challenging and time-consuming. Finally, as a purely observational approach, these at-home recording studies are not well-suited to interventions, limiting the scope of the questions that can be asked using these methods.

A third approach is to use diary or beeper-style studies (modern versions typically use text messages or emails), in which parents are asked to answer questions about their children’s activities at various intervals (e.g., Boyatzis and Janicki, 2003). These studies are, in principle, more capable of supporting interventions and can be designed to target specific types of parent/child activities (e.g., asking parents to complete a survey when their child is doing a STEM-related activity), but the data are limited and indirect (Morris et al., 2019). Asking a parent to report a child’s behavior rather than a researcher observing behavior directly often means that important nuances are being lost, and the act of filling out the diary or survey itself can distract the parent from interacting with their child.

**Figure 1:**
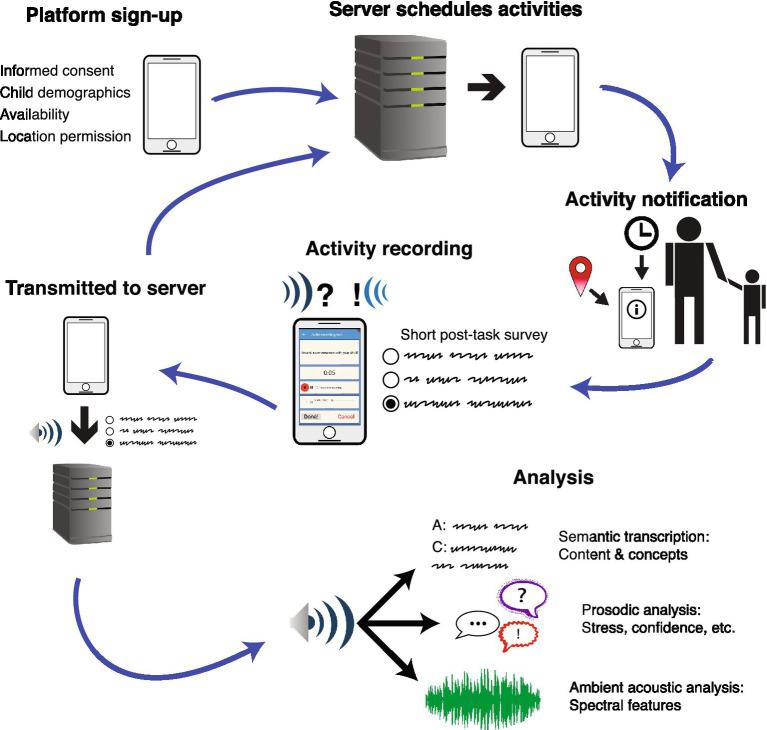
Summary of the workflow of the Talk of the Town platform, from initial sign-up to data analysis. See text for more detailed descriptions of each step.

We present a novel tool and novel approach for studying informal learning through parent–child interactions. Our tool is a research platform we call “Talk of the Town.” It consists of a mobile phone app controlled by a secure server. The basic idea of the platform is as follows: Parents download the app and sign up for a study. They then are presented with notifications asking them to record a short (~5-min) conversation with their child. These conversations can be based around specific prompts designed by the experimenter (e.g., as an intervention or experimental manipulation), or they can simply capture whatever is being discussed at that time. Parents can then answer a couple of short questions (e.g., about what they were doing during the recording), and upload the recording and their responses securely to a server where they can be analyzed. Figure 1 presents a summary of how the platform functions. This abbreviated description omits a great many important details that we will elaborate below, but it should be clear already that the goal is to capture rich, naturalistic data from a broad population^1^ in a minimally intrusive way, with a flexible tool that can be used to study many different facets of informal learning in STEM and in other domains. This tool will be deployed to the broader scientific community and is designed to be flexibly adaptable to researcher or educator needs. Because researchers and educators can choose what prompts to deploy, it can be flexibility used for researchers with whatever research question they choose to explore or it can be developed by educators, as a tool for prompting (and recording) STEM engagement.

We start by describing the mobile app component in greater detail. Just using a mobile phone app avoids many of the limitations of previous approaches. The app goes wherever the parent goes, without requiring the researcher to provide any additional hardware or specialized equipment, and so it can capture data anywhere and anytime the parent and child are together (and the parent wants to engage with the app). When parents choose to do an activity, the app presents a simple recording screen (Figure 1 includes a small screenshot from the current development build of the app). The recording screen includes a prompt which can be generic or targeted to a specific topic of conversation. The parent can then record a conversation for up to 5 min. After finishing the recording, the parent can listen to it themselves, fill out a short survey describing what they were doing, and choose to upload the recording or not. This design addresses many potential privacy and confidentiality issues: Not only must the parent deliberately engage with the task and start the recording, they can also choose whether to provide the recording to researchers or not after the fact.

However, simply having the app on hand would mean that parents have to remember to use it. Therefore, the app uses notifications to prompt parents to record conversations with their child. These notifications can be triggered by several different factors. First, they are only presented during times that the parent specifies they are able to do an activity with a child in an eligible age range, which they indicate during the onboarding process. Second, these notifications can be timed based on researcher-specified intervals and tied to specific tasks. For example, a researcher could create a longitudinal study that provides a notification to the parent at regular intervals triggered by completing the previous session (see Figure 1). This can also be used for intervention studies: The prompt for an activity can be a specific educational task, varied across participants, and researchers can examine whether that intervention has an impact on subsequent conversations. Third, the notifications can be triggered by a location, such as an exhibit at a museum, a historical statue in a park, or even the more day-to-day such as grocery stores or libraries.

The ability to link notifications to location specific sites is a unique (as far as we know) feature of the platform. “Geofences” are simply a set of GPS coordinates and a radius. The app is designed such that, if the parent provides location permissions (also part of the onboarding), if the phone detects that it is in proximity to one of a large database of landmarks provided by researchers, it will present a notification and a location-specific activity for the parent to do with their child. One can think of this as similar to some museum-based studies, with three key differences: Rather than placing a recording system in the exhibit, it is in the hands of the parents; rather than being restricting in time to just the moment of museum interactions, automated follow-ups can be delivered hours, days, or weeks later; and rather than being restricted to a handful of exhibits or a single museum, it can be applied to the entire world. While particularly useful for outdoor landmarks, we have also discussed the possibility of partnering with certain museums to use “beacons” placed in specific exhibits to use the app as an alternative to a standard museum-based study. Ultimately, the app just needs to know that it is in proximity to a potential activity location, and it can notify the parent to prompt them to engage in an activity with their child at that location, which the app can then record.

The data that the app provides also strikes a careful balance. Like home-recordings, the data that the app captures is very rich, consisting of conversations between parents and children, as well as capturing some information about the ambient environment (e.g., indoors vs. outdoors; Van Hedger et al., 2019a,b). However, because the recordings are short, parent-initiated, and tied to specific prompts, they are much more focused and require less processing to isolate relevant interactions. In addition, researchers can include short surveys for parents to fill out after the recordings, which can provide valuable metadata about what the parent and child were doing at the time, levels of engagement, and more.

The platform’s design is intended to be highly flexible for researchers or educators. These investigators only ever need to interact with the server side of the platform to develop their specific use content. The server, the Sage Bionetworks Bridge platform (Sage Bionetworks, 2022), is designed for use with medical research apps, and therefore has a level of security and encryption more than suitable for behavioral research. Investigators can specify the parameters of “assessments” on the server, such as the content of a prompt, the timing of notifications, and any geofences it should be tied to (as well as the geofences themselves). This information is then transmitted to the app any time the parents log in to the app, updating the app’s internal library of tasks and scheduling future notifications. Notably, this does not require the app itself to undergo a full update. Rather, one can think of it like a waiter at a restaurant presenting an order to the kitchen: the kitchen already has the tools and ingredients to make the dish, they just need to be combined and presented back to the customer. This means that the types of tasks available are restricted to the previously described recording activities and short surveys, but it is easy to add new studies or change existing studies on the fly.

A potential key benefit of the app is that, with careful recruitment, participants may represent a broader and more representative sample of the population than typically found in laboratory studies that tend to overdraw from higher SES populations in university and city centers (Fernald, 2010). Because anyone with a mobile phone can sign-up to engage with a study run on the app, families can participate without concern of transportation into a lab or being locally-university based. Indeed, studies comparing online data collection to in-lab collection has already revealed better representation through online methods that require computers (e.g., Scott and Schulz, 2017). We believe that by further lowering barriers (users without computers, who have phones can participate), we will be able to achieve even greater representation. Furthermore, additional languages can be relatively easily available, and we are looking to develop a Spanish version in the next year. Finally, we note that labs can offer financial incentives to participating families, which may increase participation from under-resourced communities.

## 2. Challenges and pitfalls

Naturally there are challenges that come with the development of any new technological tool. In the case of a mobile-app-based platform like this one, the first challenge is the development and maintenance of the app itself. Fortunately, because the functions of the app itself are relatively simple, the first author was able to do most of the initial development in the Flutter framework (Google, 2022), but professional assistance is required to polish the app to the point that it can be released on an app store, which is a moderate investment. However, initial development is not enough. Every new version of iOS or Android operating systems, or updates to the code libraries that support critical functions like notifications or sound recording, will require some degree of maintenance work on the app itself at regular intervals, and in turn require a consistent investment of resources. The plan is to make the app itself open-source, so that other research groups can create their own “clones” of the app, but this should not be undertaken lightly. Mobile apps, in general, require a greater degree of development effort than a web-based app (e.g., Chan et al., 2017; Webster et al., 2017).

The second challenge is recruitment and retention of participants. In conversation with other groups that have attempted app-based research in the past (see acknowledgements), this has consistently surfaced as a major challenge. To get parents to download an app and continue to use it, the app must offer the parents something in return. That can take the form of compensation or prizes for their children, but ideally *engaging with the app itself* should be valuable to parents. We intend our use of the platform to present fun activities for parents to do with their children, such as scavenger-hunt-like activities at zoos, museums, and parks using the geofencing system, as well as education-focused activities as part of research studies, but it will be up to individual investigators to consider how they may choose to recruit users. Since the primary unit of activity in the app is a parent/child interaction, we want to make those interactions fun and rewarding for both parents and children, and by doing so, we hope to collect a great deal of data simply because it is something parents *want to do*.

However, after surmounting the challenges in launching the app and getting families to participate, there is a third challenge to consider: processing the data. If the app is very successful, vast amounts of rich audio data will require processing. The richness of parent/child conversations is certainly a boon from a research standpoint, but practically speaking it is also a burden. To manually transcribe hundreds or thousands of recordings, even short ones, requires thousands of person-hours and is difficult work. To get ahead of this problem we have been developing a partially automated data-processing work-flow, completely divorced from the app and platform itself. To keep the app simple, all it does is upload the data. Once we have the data in-hand, we have developed a workflow that starts with an automated transcription.^2^ The transcript produced by this system is then imported into ELAN (2022) for human-proofing, which in early pilot runs has taken half the time or less than transcribing from scratch. These tools will also be available to promote Open Science. This is certainly not the only solution to the problem, but any lab that intends to collect this kind of rich data on a large scale needs to be ready to process it, and it is a non-trivial problem.

## 3. Unique research opportunities

Why is it worth going through all this trouble? In short, because the Talk of the Town platform provides a ground-breaking opportunity to study informal learning in greater depth and with a broader population than ever before. The app’s simple design nonetheless supports substantial flexibility, thanks to the ability of the server to specify content, target populations, and track completion of specific tasks. Using the platform it is possible to conduct experience sampling studies, longitudinal studies, and simple intervention studies. With the geofencing system, truly unique types of studies that capture, naturally, when and how families interact with certain landmarks in their environment can be carried out, targeting learning in time and space. The data that will be captured by the app will ultimately form a corpus of recordings connected to demographic, timing, and location metadata that will provide opportunities for re-analysis on a variety of issues. Semantic content, prosodic content (including vocal stress markers), and ambient acoustic environment are accessible to researchers. Furthermore, the app offers the potential for valuable collaborations with zoos and museums all over the world without requiring prohibitive investment of space, money, or time by these sites nor by principal investigators.

Finally, from a practical standpoint, professionally developed apps cost hundreds of thousands to millions of dollars, and developing a secure server from scratch would be no less expensive. The app uses open-source tools and only required advanced but amateur programming skills to develop to the point of basic functionality.^3^ While some professional development will be needed to polish and improve the app in the future, it should not require full-time professional attention to maintain. As for the server, using external options like the Sage Bionetworks Bridge is not free, but the cost of an annual research agreement is far preferable than the time and money investment required to build such a system from scratch. In particular, being able to lean on others who have experience handling sensitive medical data makes the regulatory aspect much easier to deal with, even when capturing personally identifiable data from a vulnerable population. The data recorded by the app are encrypted and transmitted securely to a heavily-protected server overseen by professional security staff.

## 4. Conclusion

The Talk of the Town platform is an example of a new kind of research tool that will be invaluable for understanding the role of informal learning in STEM education and success. While we hope to make our specific platform available to a broad range of collaborators, we hope that our description of its development and underlying systems will also inspire other groups to build on our efforts and develop their own tools to address specific questions or issues. The potential of mobile-app-based research for the science of learning is tremendous, but the successes to date have been few and relatively costly in time, effort, and expertise (e.g., Morris et al., 2019). It is easy to see why this kind of research is not yet widespread, given the enormous up-front investment and perpetual challenges of recruitment, retention, and maintenance. However, it is our belief that a single successful broad deployment of a platform like Talk of the Town could yield such a bounty of novel and valuable insights into informal learning through parent–child interactions that it will open the floodgates to a whole new generation of research programs on informal learning more broadly, and perhaps revolutionize how researchers approach the science of learning as a whole.

## Author contributions

JK drafted the manuscript and created the figure. JK, IB, PS, and EB revised and edited the manuscript. All authors contributed to the article and approved the submitted version.

## Funding

This work was supported by a Templeton Foundation Developing Belief Network postdoctoral fellowship to JK and NSF EAGER grant 2121842 “Science of Learning: ‘Talk of the Town’ app development” to JK, IB, PS, and EB.

## Conflict of interest

The authors declare that the research was conducted in the absence of any commercial or financial relationships that could be construed as a potential conflict of interest.

## Publisher’s note

All claims expressed in this article are solely those of the authors and do not necessarily represent those of their affiliated organizations, or those of the publisher, the editors and the reviewers. Any product that may be evaluated in this article, or claim that may be made by its manufacturer, is not guaranteed or endorsed by the publisher.

## Footnotes

^1^The obvious equity-limiting factor of this platform is that only families with smartphones are able to use it. However, smartphones are increasingly ubiquitous (more than 85% of Americans now own a smartphone, Pew Research Center), and future development can ensure compatibility with older phone models.

^2^To go into some detail, after exploring several options we found that (at the time of this submission) Microsoft’s Azure Speech-to-Text system was better at handling children’s voices than most of the alternatives, which is naturally a critical concern for our purposes. Furthermore, because our institutions have an agreement with Microsoft, we are able to run this service in a locally-hosted "container," satisfying privacy and confidentiality rules.

^3^Admittedly not quickly; it took about a year and a half to develop.
